# Bioinformatics Goes Viral: I. Databases, Phylogenetics and Phylodynamics Tools for Boosting Virus Research

**DOI:** 10.3390/v16091425

**Published:** 2024-09-06

**Authors:** Federico Vello, Francesco Filippini, Irene Righetto

**Affiliations:** Synthetic Biology and Biotechnology Unit, Department of Biology, University of Padua, 35131 Padua, Italy; federico.vello@studenti.unipd.it (F.V.); irene.righetto@bio.unipd.it (I.R.)

**Keywords:** bioinformatics, computational biology, bio-databases, data mining, virus–host interaction, sequence alignment, phylogenetics, phylodynamics, virus evolution

## Abstract

Computer-aided analysis of proteins or nucleic acids seems like a matter of course nowadays; however, the history of Bioinformatics and Computational Biology is quite recent. The advent of high-throughput sequencing has led to the production of “big data”, which has also affected the field of virology. The collaboration between the communities of bioinformaticians and virologists already started a few decades ago and it was strongly enhanced by the recent SARS-CoV-2 pandemics. In this article, which is the first in a series on how bioinformatics can enhance virus research, we show that highly useful information is retrievable from selected general and dedicated databases. Indeed, an enormous amount of information—both in terms of nucleotide/protein sequences and their annotation—is deposited in the general databases of international organisations participating in the International Nucleotide Sequence Database Collaboration (INSDC). However, more and more virus-specific databases have been established and are progressively enriched with the contents and features reported in this article. Since viruses are intracellular obligate parasites, a special focus is given to host-pathogen protein-protein interaction databases. Finally, we illustrate several phylogenetic and phylodynamic tools, combining information on algorithms and features with practical information on how to use them and case studies that validate their usefulness. Databases and tools for functional inference will be covered in the next article of this series: *Bioinformatics goes viral: II. Sequence-based and structure-based functional analyses for boosting virus research*.

## 1. Bioinformatics: From Pioneering Tools to Current, Reliable Toolbox

### 1.1. Database and Tool Design: Genomics Make Them Bigger and Better

Although the origin of bioinformatics can be traced back several decades, the performance of the initial and pioneering methods was sometimes poor as the DNA and protein sequence sets available then were still limited. Due to this limitation, the analysis and prediction algorithms had to be based mainly on statistical considerations and/or on the evaluation of physicochemical properties [1]. Then, the development of efficient gene cloning, polymerase chain reaction (PCR) [2] and DNA sequencing methods opened the route to the genomic era, and a great boost to bioinformatics came from both (i) the decision by scientific journals to make it mandatory to deposit new sequences in public databases, prior to publication [3] and (ii) the advent of the World Wide Web, along with graphical user interfaces (GUIs), which started the online sharing of databases and tools, hosted on the servers of bioinformatic organisations and/or research labs. 

The exponential growth of publicly available sequence data was further favoured by the development of cheaper and faster sequencing methods, collectively referred to as Next Generation Sequencing (NGS). Using NGS, millions of DNA fragments are sequenced simultaneously, providing insights into genome structure, genetic variations, gene expression profiles, and epigenetic modifications [4]. Bioinformatic methods have been crucial for overcoming some technical issues in sequencing technologies for viral genomes, as NGS-based discovery of viral sequences in mixed samples remains a challenging task (most analysis steps are not easily automated) [5]. 

In recent decades, the availability of a huge number of sequences has strongly enhanced evolutionary analyses [6] and comparative genome annotation [7]. Furthermore, it has changed the design of bioinformatics tools, with scoring systems behind their analytical and predictive capabilities progressively shifting from statistical and theoretical considerations to large-scale empirical observation and comparison of features associated with specific gene classes and proteins, domains, etc. [8].

The “omics” era provided the most famed and large bio-databases with hundreds of thousands of nucleotide and protein sequences: Genbank, managed by the American National Council for Biotechnology Information (NCBI) [9], grew from roughly 100,000 DNA sequences in 1993 up to current 250 million ones [10]. A parallel (and coordinated, because of a long-lasting sequence exchange agreement) growth concerned both the DNA sequence database of the European Molecular Biology Laboratory (EMBL) and the UniProt database, managed by the European Bioinformatics Institute (EBI) [11]. In addition, large-scale transcriptomics, proteomics, structural genomics and interactomics international initiatives have strongly improved the annotation of most such entries by providing cross-links to expression data at both RNA and protein levels and to many protein structures and experimentally validated high-throughput evidence on protein-protein interactions. Of course, the availability of such a large number of annotated sequences prompted the creation of special databases focusing on specific genes, proteins, protein domains or organisms, including viruses. 

### 1.2. From Big Data to Huge Data: Exploring the Planetary Virome in the Petabase Era

From a historical point of view, virologists focused most of their studies on viruses causing pathologies in humans or animals, using serological approaches. However, evidence that viruses can store and transfer genetic information from their hosts, hence affecting bio-geochemical cycles, prompted further studies on virus evolution and their ecological role. Virology studies have benefited from genome sequencing technologies and tools for managing big data, but in recent years, the exponential growth of available sequence reads has led to the transition from big to “huge data”, now measured in petabase (1 × 10^15^ bases) terms. Therefore, while improving discovery and comparative analysis, paradoxically, this growth also raises some problems. Indeed, contrary to sequencing (more affordable thanks to NGS technologies), sequence analysis remains computationally expensive, especially when the assembly of contigs from short reads is performed. To address this point, petabase-scale sequence alignment methods have been developed to enhance virus discovery and access to the planetary virome. Public databases are rich in viral DNA sequences because, in addition to intentionally sequenced ones, often viral sequences are captured as contaminating sequences in samples collected for other purposes. In 2022, Edgar et al. published the development of Serratus, which is defined by authors as “a free, open-source cloud-computing infrastructure optimised for petabase-scale sequence alignment against a set of query sequences” [12]. This infrastructure is powered by up to 22,250 CPUs, allowing Serratus to process data hundreds of times faster than standard methods. 

Serratus toolkit is freely available [13], and all data are publicly available within 24 h of generation because the developers adhere to the Bermuda Principles (set out originally by the Human Genome Project). Serratus, in turn, re-analysed public data from the NCBI Sequence Read Archive database [9], screening 5.7 million ecologically diverse sequencing libraries or 10.2 petabases of data. In addition to being of great help to virus discovery and classification (or re-classification), the main aim of this project is to provide the scientific community with access to the planetary virome, as this is of pivotal importance to any global surveillance programme and readiness with future pandemics [13].

### 1.3. From Sequence Alignment to Evolutionary Studies and Functional Inference

In addition, to help assemble and annotate genomes and proteomes, sequence alignment methods are crucial to both evolutionary and functional studies. Over the past two decades, BLAST (Basic Local Alignment Search Tool) [14] has been—and still is—the most used bioinformatic tool worldwide, and so far, many applications have been developed based on its algorithm. Nonetheless, several users, from university students to senior researchers, are still surprisingly unaware of the great discovery potential of BLAST (and, in general, of sequence alignment) when properly handled by an expert user. While the “tricks” and “hidden treasures” for functional analyses will be covered in paper II of this series, some general points of interest will be discussed below. 

Although each BLAST application allows users to direct BLAST execution to a specific database (among many made available in the database selection menu), most users do not change the default settings. Consequently, the sequence query is compared to the non-redundant (nr) nucleotide/protein database, and this often represents an unnecessary waste of time on the client side and computational power on the server side. Furthermore, this can result in misleading or missing information, with relevant results buried by noise (e.g., many hypothetical sequences not validated by evidence) or by the fact that the default of showing only the first 100 results is unintentionally retained. Most BLAST users are unaware that this limitation is set by default on the NCBI BLAST web server and that this value can be increased. Therefore, extracted sequences should only be referred to as “BLAST results” when <100 are displayed. Instead, when 100 results are listed, many other sequences are most likely not. For example, if relevant subject sequences started after the 120th hit, the user would be missing (not seeing) the most important information from the BLAST run.

Furthermore, most homology searches are carried out using only the basic applications (nucleotide BLAST and/or protein BLAST), missing additional (and perhaps crucial) information that can be recovered using the “translated” versions (BLASTx, tBLASTn, tBLASTx) versions [15].

Of interest to virus research, BLASTx accepts a nucleotide sequence query for scanning protein databases. To achieve this task, amino acid sequences generated by dynamic translation of that nucleotide query in all six reading frames (i.e., including three from the reverse complement sequence), in turn, become query sequences of multiple BLAST runs against the selected protein database. In this way, all proteins encoded by separate and/or overlapping genes of both strands of a newly sequenced virus genome can be revealed. Furthermore, when using the host proteome as a reference database, any protein regions of virus-host homology are also shown.

tBLASTn does the reverse work, i.e., a protein sequence query is launched against (all six-frame translations of) nucleotide databases. In the virology community, this can help identify “hidden” orthologues of a viral protein of interest. Indeed, when using BLASTp, the query protein sequence is compared only with sequences already deposited as proteins in the database on the date of the BLAST run. Instead, tBLAStn can identify additional proteins among (i) translations from newly sequenced genomes (not yet translated and deposited in proteome databases) and (ii) putative proteins that have escaped large-scale automatic computational inference.

Last but not least, tBLASTx is of particular interest for virus evolution studies when the idea behind the project is to give special weight to protein-coding genes and their functions. Nucleotide sequence comparisons are largely used in evolutionary studies. However, nucleotide BLAST gives the same weight to missense mutations, which involve an amino acid change, and to silent mutations that transform one codon into another for the same amino acid (no change in the protein sequence). Instead, tBLASTx compares a dynamically translated nucleotide query sequence to the genomic/transcriptomic database (or given dataset/sequence), thereby eliminating silent mutations and performing a comparison based on amino acid changes and substitution matrices.

Regardless of the alignment tool used, be it BLAST or another local or global alignment method, the comparison is carried out in “pairwise” mode between a query sequence and each sequence of the selected dataset/database [16]. Hits showing similarity across the entire sequence, or only in specific parts of it, can provide hints about putative characteristics of the analysed sequence. At the same time, a BLAST search can provide a collection of homologous sequences, which can subsequently be used to construct a multiple alignment (MSA). 

MSAs are fundamental for both evolutionary and functional studies because only a rich and balanced set of aligned sequences can properly reveal the level of local conservation and divergence. The collection of large datasets and their taxonomic and/or subclass balancing has been greatly favoured by the “big data” generated from NGS. This, in turn, improved the smart design of experiments, allowing us to infer in silico which conserved residues might be crucial for function and where functional modulation occurs.

It should be emphasised that, for genes encoding proteins, the combination of homology searches with DNA and amino acid sequences is more informative than analyses carried out on a single type of sequence. Indeed, when using DNA sequences, the overall evolutionary divergence is best assessed by identifying synonymous mutations, which are hidden by amino acid sequences. Instead, the analysis of protein sequences is more relevant for functional inference since the burden of silent mutations is eliminated while variation in amino acid properties is evaluated.

The progressive growth of sequence datasets also allows us to define more and more position-specific score matrices (PSSM), e.g., via PSI-BLAST [17], as fingerprints specific to different, conserved structural domains and functional regions. Indeed, the recognition of domains and regions within proteins can facilitate the understanding of evolutionary pathways and, in particular, the co-evolution of viral and host proteins in the context of host-pathogen interactions. 

A commonly accepted assumption is that a complete protein sequence follows a single evolutionary trajectory. However, since it is well known that a domain can exist in various contexts, this is not valid for multi-domain proteins [18]. For this reason, it is very useful to associate the results of homology searches with information on the domain architecture of the analyzed protein, as it happens, e.g., with the BLAST NCBI graphic interface, which shows, together with the list of hits, the result of the Conserved Domain Database (CDD) scanning. CDD is an MSA-derived collection of models of protein domains and protein families, capable of providing domain architecture annotation for translated proteins and nucleotide sequences and a basis for assigning gene product names and putative/predicted functions [19]. 

Hidden Markov models (HMMs) and machine learning-based methods are increasingly used for the definition of domain profiles and functional analyses, but these will be covered in the second review of this series, which also will cover MSA-derived functional markers such as regular expressions (patterns) and domain profiles, as well as structural bioinformatics (protein structure databases, structural modelling by homology, fold recognition or ab initio methods, structure superpositions, docking simulations, molecular dynamics, etc.).

Section 2 of this article focuses on sequence databases dedicated to pathogenic viruses and those with broader taxonomic representation while still being of interest to virus research. Special emphasis is given to virus-host interactions. In Section 3, phylogenetics and phylodynamics tools are discussed.

## 2. Bio-Databases

Current tools and technologies produce an enormous and growing amount of data, both in terms of virus genome/transcriptome sequences and details of virus-host interactions. This data are stored in both dedicated and general databases for retrieval, visualisation, and analysis. The recent pandemics of SARS-CoV-2 (severe acute respiratory syndrome coronavirus) and MERS-CoV (Middle East respiratory syndrome coronavirus), as well as the spread of H1N1 and H5N1 influenza viruses, Ebola, Zika and West Nile viruses, have highlighted the focus on the (re)emergence of zoonotic viruses. These events, which arise because of ecological and environmental changes, can lead to devastating blows in economic, health and social interaction terms. Therefore, it is a priority to focus efforts on two activities: improving therapeutic measures and strengthening surveillance to manage future epidemics. 

In such a scenario, the value of databases in sharing virus data is fundamental: integrating them with clinical data was (and is) fundamental to tackling diseases and mitigating their effects [20]. Focusing on PPIs (protein–protein interactions) is of primary importance because they play a fundamental role in the development of vaccines and therapies, as they are involved in viral replication and immune escape [21]. 

### 2.1. Sequence Databases

#### 2.1.1. Open-Source Databases

Open-source databases are coordinated by the International Nucleotide Sequence Database Collaboration (INSDC) [22], including three member organisations: (i) Research Organization of Information and Systems—National Institute of Genetics (ROIS-NIG), corresponding to the DNA Data Bank of Japan (DDBJ), (ii) European Molecular Biology Lab—European Bioinformatics Institute (EMBL-EBI) and (iii) National Library of Medicine—National Council for Biotechnology Information (NLM-NCBI) at the American National Institutes of Health (NIH). INSDC Members provide data resources that include raw sequence reads and alignments, structured metadata describing investigated samples such as taxonomic information, experimental and project design, assembled nucleotide sequence data with functional annotation, and sequence-derived analyses. All such data are freely accessible to the scientific community thanks to the continuous communication, data exchange and interoperability among these organisations. 

As reported [23], DDBJ is a public database collecting nucleotide sequences, study, and sample information and distributing them without access restriction. Notably, during the COVID-19 pandemic, the DDBJ Centre openly shared SARS-CoV-2 genome sequences. In October 2020, the DDBJ Centre activated a new public repository called MetaboBank for experimental raw data and metadata from metabolomics research [24].

The European Nucleotide Archive (ENA) [25] is maintained by the EMBL-EBI [11]. In the context of the INSDC collaboration, ENA analyses, archives, and disseminates sequence data ranging from raw reads to complete assemblies, which are functionally annotated, enriching the data with contextual information on samples and experimental configurations. 

In the last overview of ENA services, the authors focus on three work areas: FAIRness of ENA data (where FAIR stands for Findable, Accessible, Interoperable, and Reusable), pandemic preparedness, and foundational technology [26]. In particular, in order to support genomic surveillance efforts even after the end of the COVID-19 emergency, ENA continued to provide SARS-CoV-2 data mobilisation, which has been adapted for broader pathogen surveillance.

Of special interest to the virologist community, ENA hosts a web-portal called Pathogens [27], which includes nucleotide sequences, raw genomic data, sample metadata, and relevant scientific literature about HIV, influenza, Hepatitis B, *Plasmodium falciparum* and lesser-known pathogens affecting humans, such as the mammarenavirus Lassa virus (LASV). This resource also reports information on pathogens that affect other animals. The goal of this database is to integrate additional data types, including protein sequence, structure and chemistry data from other public data resources. EMBL highlights the relevance of the free accessibility of Pathogens, as this portal is fully open access and FAIR.

In addition to the ENA nucleotide database, EMBL-EBI also manages protein sequences deposited in the UniProt knowledgebase [28]. UniProt is the world’s leading resource of comprehensive, high-quality, and freely accessible sets of protein sequences annotated with functional information [29]. The number of sequences in UniProtKB has risen to over 227 million in 2023, and when using the “virus” keyword as a search query, more than 7 million entries were found by the end of May 2024. UniProt entries are deposited in two subsections: reviewed entries with detailed annotations from experimental evidence and/or the literature are deposited in SwissProt, which was originally created and curated by the Swiss Institute for Bioinformatics at the ExPASy server [30]. Unreviewed entries are deposited in the TrEMBL subsection. 

To give readers an idea of the current ratio of reviewed to unreviewed entries, of the more than seven million “virus” records mentioned above, only fewer than 29,000 belong to the SwissProt subsection. This means that users should be aware that most sequences are unreviewed and provided by automated systems using a variety of machine-learning techniques. While these methods undergo progressive improvements, they are not error-free, and when merging incorrectly assembled or annotated sequences into a dataset, this can result in misleading information.

The American NCBI manages a huge amount of biological information, including the well-known PubMed portal for consulting scientific literature, via the Entrez browser, which provides search and retrieval operations for data from 34 distinct databases [31]. At the end of May 2024, browsing with Entrez “All databases” on the NLM page [32], and once again using “virus” as a keyword, the positive hits from 32 databases are sorted into the following six sections: Literature, Genes, Proteins, Genomes, Clinical, and PubChem. Each section is, in turn, divided into subsections, which can be consulted individually. In the Literature section, in addition to approximately 1.5 million articles from PubMed, the keyword “virus” retrieved >30,000 books and >2000 MESH terms. Of course, not all positive results are 100% related to viruses; for example, some books from the Bookshelf subsection deal with a more general topic, such as pathogens or causative agents of a disease, etc., while others focus specifically on viruses. 

The PubChem section reports very useful information on antivirals in the following subsections: BioAssays (69,479), Compounds (1832), Pathways (2286), and Substances (2906), where numbers in brackets concern positive results for the keyword “virus”. Further information of interest to clinical virologists can be found in the Clinical section, including, e.g., subsection ClinVar, which collects > 2000 clinical variants associated with resistance or increased sensitivity to specific infections. Sections Genes, Proteins and Genomes collect several million gene and protein sequences as well as complete viral genomes; moreover, the GEO DataSets and GEO Profiles subsections allow access to records in the Gene Expression Omnibus (GEO) repository [33]. Open access datasets deposited in GEO hold great value for knowledge discovery, particularly when integrating “static” information on genome sequence with gene expression profiles. Effective June 2024, NCBI’s Assembly resource will no longer be available, as NCBI Assembly data can now be found on the NCBI Datasets genome pages.

Together with virus sequences deposited into the general databases Genbank [31] and RefSeq [34], NCBI hosts NCBI Virus, a community portal for viral sequence data from RefSeq, GenBank and other NCBI repositories [35]. NCBI Virus accepts queries by sequence (hence starting a BLAST run) or by virus name or taxid [36]. Moreover, the NCBI FLU Database, also named Influenza virus Resource [37], is entirely dedicated to the Influenza virus with many applicable parameters to select the area of the world from which the samples and respective sequences originate. From fall 2024, this resource will be redirected to NCBI Virus. The FLU database also allows you to build a phylogenetic tree including up to 1000 selected sequences, using different statistical approaches. 

As highlighted by Zhao and co-workers [38], public databases are a cost-effective method for practising pathogen outbreak surveillance. At the time of writing their article (winter 2021), the authors reported statistics on viral sequences deposited in the NCBI virus database. More than half a million records were analyzed covering >24,000 viruses from 240 countries/regions, finding that the most widely distributed viruses are influenza A virus, human immunodeficiency virus 1, hepatitis B virus, rabies lyssavirus, dengue virus and measles morbillivirus.

A family of database resources aimed at supporting global academic and industrial communities is made publicly accessible by the National Genomics Data Center (NGDC), which is part of the China National Center for Bioinformation (CNCB) [39]. Precious sequence data from viruses and other organisms, not available elsewhere, can be found at the Genome Sequence Archive (GSA) website [40], which is the first repository of genome sequence data with international journal recognition in China. In the past two years, considering the global threat of the monkeypox virus and SARS-CoV-2, CNCB-NGDC has a newly constructed monkeypox virus resource, also taking care of frequent updates of the SARS-CoV-2 genome sequences, variants and haplotypes [41].

A database was developed in 2022 at Tongji University, Shangai, China, focusing on human disease-related Virus Mutations, Integration sites and Cis-effects (ViMIC), providing users with information on >31,000 virus mutation sites, >105,000 viral integration sites and >16,000 viral target genes [42]. Furthermore, the ViMIC databases collected from the public domain information on more than 1 million virus sequences of eight viruses in almost 80 human diseases. ViMIC users can also explore cis-effects of virus–host interactions, like, e.g., histone modifications, binding of transcription regulators and chromatin accessibility [43].

A multi-national effort headed by researchers from Tsinghua University, Beijing, China, constructed in 2017 a virus database called VirusDB, including an online inquiry system for viral classification and prediction [44]. The classification information of the single/multiple-segmented viral reference sequences present in VirusDB is downloaded from NCBI. When submitting genome data in FASTA format, prediction results with the five closest neighbours and their classifications are returned by email [45].

Influenza Virus Database (IVDB) [46] was established in 2007 by the Beijing Institute of Genomics (BIG) to integrate information and create an analysis platform for genetic, genomic, and phylogenetic studies of influenza viruses (IV). IVDB hosts complete genome sequences of the influenza A virus generated by the BIG and curates all other published IV sequences after expert annotation. To facilitate analysis of global viral transmission and evolution, the IV Sequence Distribution Tool (IVDT) was developed to display the worldwide geographic distribution of chosen viral genotypes and to couple genomic data with epidemiological data [47].

Although several resources for HIV-related research have been created in recent decades, which would deserve a full dedicated paper, as an example, we can cite here the Los Alamos HIV databases [48]. This portal provides users with updated information available in a selection menu with eight specific sections: Sequence DB, Immunology DB, HXB2 Feature DB, Env Feature DB, Neutralisation DB, HCV DB, HFV DB, COVID-19 Genome Analysis Pipeline. In addition to a search menu, a large number of online tools are made available for purposes as diverse as alignment, annotation, consensus building, genome navigation, motif scanning, peptide mapping, heat maps, epitope analysis, etc. [49].

A knowledge resource to understand virus biology and diversity is ViralZone [50], hosted by the ExPASy server of the Swiss Institute for Bioinformatics (SIB) [51]. ViralZone has a nice and user-friendly graphical interface. Buttons at the top of the home page direct subsequent searches to the DNA, RT, or RNA virus pages. While the central part of the home page is dedicated to the latest news (updated or new website features, novel datasets, etc.), the left side menu allows you to navigate in five main sections (Taxonomy, Biology, Hosts, Genomes, Resources), each with subsections with active links redirecting to related information pages. 

Most recent ViralZone statistics (May 2024) show that virus description pages concern 158 viral Families and 711 Genera while linking to (UniProt release 2024_02) 10,530 reference proteomes, 17,390 manually reviewed proteins and 5,467,967 unreviewed proteins.

#### 2.1.2. Registered Users Databases

GISAID is short for Global Initiative on Sharing Avian Influenza Data [52]. It was established in 2006 due to the global spread of H5N1 avian influenza [53], and due to the 2020 pandemic, it became the most popular SARS-CoV-2 repository. At the time of last access to this database [52], 16,760,454 hCoV-19 (human CoV-19) genomic sequences had been deposited. Although GISAID’s strength lies in rapid data sharing, access to such data is only permitted once an account is created, so scientists using GISAID must bear the terms of use in mind. 

Many academics have highlighted the lack of transparency of this platform because (i) the sequences are not “open access”, (ii) access for different users seems to be arbitrary and (iii) GISAID appears to be unfair in applying penalties to users who do not properly comply with the terms of use [54]. After logging in, different areas are available: EpiFlu™ (influenza virus), EpiCoV™ (SARS-CoV-2), EpiPox™ (MPXV), EpiArbo™ (Arboviruses). Clicking on these areas, the user can retrieve the genomic sequences of interest. 

Data from GISAID are employed in other tools, such as NextStrain [55,56], Microreact [57,58], CoVsurver [59], FluSurver [60] and COVID-19 Genome Tracker [61,62].

BV-BRC is a registered user resource [63] with Bacterial and Viral (BV) information from the Bioinformatics Resource Center (BRC), which in turn is a programme of the American National Institute of Allergy and Infectious Diseases (NIAID). 

BV-BRC was created by merging the PAThosystems Resource Integration Center (PATRIC), the Influenza Research Database (IRD) and the Virus Pathogen Database and Analysis Resource (ViPR) to help researchers analyse viral and microbial genome sequence and omics-related data. BV-BRC provides a unified interface for sequence and data mining and a powerful suite including several command-line bioinformatic tools for bacterial and viral research [64].

### 2.2. Protein-Protein Interaction (PPI) Databases

Although several valid reviews of virus databases have been published so far, even in recent years, the focus has often been on classification rather than interactions. This is surprising since the very existence of viruses—whose status is that of obligate parasites—depends on interactions (with the host cell). Indeed, viral particles, by themselves, are simply macromolecular complexes containing genomic information because until they infect a living host cell, viruses can neither replicate their genomes nor produce components to generate further viral particles. Early infection is mediated by the interaction of a viral capsid protein (such as the hemagglutinin of influenza viruses or the spike protein of coronaviruses) with functional receptor(s) exposed on the surface of the host cell and made up of glycan moieties (such as sialic acid) or proteins. Soon after infection, further interactions occur, where viral components hijack intracellular host machinery to drive the production of novel viral particles. Therefore, PPI databases are of fundamental interest to many virologists interested in deepening their knowledge of mechanisms underlying infection and in designing and developing specific antivirals. 

The interactions among human and viral proteins can be analysed thanks to wet-lab technologies, or through computational approaches. The experimental approach can be mediated by low-throughput (i.e., surface plasmon resonance, isothermal titration calorimetry, pulldown assays) and high-throughput (two-hybrid system and affinity purification-mass spectrometry) detection systems. 

Although recent advances in high-throughput techniques have made an important contribution to the discovery of the human-virus interactome, the data obtained from these experimental technologies are not sufficient to shed light on the entire picture [65].

On the other hand, in silico investigation on protein domain structures and linear/structural motifs can give a strong boost to functional analyses and technological design, aiding experimental efforts. One such example is the contribution of these cost-effective forecasting methods in the fight against epidemics of new viruses (e.g., SARS-CoV-2): the design of vaccines and drugs could be started without delay [65]. Studying surface characteristics such as hydrophobicity and electrostatics is critical to understanding virus–host PPIs [66,67], as even subtle variations in such characteristics can modulate interactions with host cells and immune recognition, thus representing a possible fingerprint for pathogenicity shift [68].

Virus–host PPI databases belong to two groups: generic PPI databases that also contain virus–host interaction information and databases of PPIs for a specific virus or for those viruses that infect a particular host [69].

#### 2.2.1. Generic PPI Databases

##### BioGRID

Established in 2006, BioGRID (Biological General Repository for Interaction Datasets) is a manually curated, open-source database hosting protein, genetic and chemical interactions from different species [70]. Data stored in this database, coming from experimental evidence published in peer-reviewed literature, can be used in the biomedical field, particularly for human health and disease. Chemical interactions concern protein or gene interplays with small molecules. 

BioGRID presents some extensions, such as cellular regulations, ORCS (Open Repository of CRISPR Screens) and specific disease areas. Since the 2020 pandemic, BioGRID has housed protein identifiers for SARS-CoV-2, SARS-CoV and MERS-CoV to help identify drug targets and lead compounds [71]. Interaction datasets can be visualised using Cytoscape [72,73], NDEX [74,75], and esyN [76,77]. 

Coronaviruses are not the only viruses present in the BioGRID database: clicking on BioGRID statistics, interactions for different viruses (e.g., Hepatitis C Virus, Human Herpesvirus 1, 2, 3, 4, 5, 6A, 6B, 7, 8, Human Immunodeficiency Virus 1 and 2, Human papillomavirus 10, 16, 32, 5, 6b, 7, 9, Simian Immunodeficiency Virus, Simian Virus 40, Tobacco Mosaic Virus, Vaccinia Virus) are reported.

##### IntAct

IntAct (managed by the EBI team) is an open-source, freely available PPI database containing data derived from both the scientific literature and direct data depositions [78]. Two levels of annotation characterise this resource: one from the International Molecular Exchange (IMEx) consortium and the other one from MIMIx [79]. IntAct contains several datasets, including the Coronavirus interactome [80,81]. Here, data come from peer-reviewed publications and from preprints if of interest. 

The Complex Portal [82] offers interactions of proteins with small molecules, nucleic acids and polysaccharides (glycosaminoglycans). The IntAct web site is user-friendly and provides two levels of data access: the quick search and the batch search. Combining simultaneous query terms, the latter offers a result refinement. Outputs can be retrieved and downloaded in graphical and tabular format [81]. 

Recently, IntAct was used (along with similar tools) for the construction of a human protein–protein interactome and drug–target network to screen for drugs against the SARS-CoV-2 host factors [83].

##### MINT

The MINT database [84] is a public database designed to store information about protein interactions [85]. Viral proteins come from relevant pathogens: papilloma viruses, human immunodeficiency virus 1, Epstein–Barr virus, hepatitis B virus, hepatitis C virus, herpes viruses and Simian virus 40. PPIs are manually curated from the literature or imported from other databases, e.g., IntAct and HIV-1 Human Protein Interactions Database. 

Collecting all protein interactions between viral and human proteins from MINT, a virus-specific database named VirusMINT has been created, storing (at the date of publication [86]) over 5000 interactions involving almost 500 unique viral proteins from >110 different viral strains. Results from the VirusMINT queries are displayed with a user-friendly graphical viewer.

#### 2.2.2. Virus–Host PPI Databases

##### VirusMentha

VirusMentha is a freely available virus–virus and virus–host interaction resource [87,88]. Redundancy is removed weekly through synchronisation with the IMEx databases, providing integration of virus-host interactions. VirusMentha’s strongest point lies in its ability to detect all published virus–host interactions, unlike other resources focused on specific organisms, such as, e.g., HCVpro (see below) or the HIV host–interaction map [89,90]. 

Virus Mentha integrates different databases: MINT, IntAct and BioGRID (see above), DIP [91], and MatrixDB [92]. VirusMentha has been crucial in shedding light on the interactions of 14-3-3 proteins with viruses [93]. This family of ubiquitous and exclusively eukaryotic proteins has an enormous number of binding partners, as it is involved in a wide range of general and specific signalling pathways, suggesting that it plays a critical role in health and disease. This work [93] suggests interactions among 14-3-3 β, θ, ε, γ, η, ζ isoforms and single-stranded RNA viruses (ssRNA) such as the influenza A virus (IAV), measles virus, human respiratory syncytial virus, human immunodeficiency virus (HIV), La Crosse virus, and double-stranded DNA (dsDNA) viruses like herpes simplex virus type I, human herpes 4, hepatitis B virus (HBV), Nipah virus, Hendra virus and Murid herpes virus.

##### VirHostNet

VirHostNet is an open and gold standard knowledgebase dedicated to virus-virus and virus–host PPIs [94]; its updated release is version 3.0 [95]. Access to data and output visualisation is performed by a user-friendly web interface based on Cytoscape. The goal is to model host/virus interactome using an undirected graph for data exploration and examination. 

Data (e.g., virus/host PPI network) can be retrieved in different ways, of which the first one is the quick search, where UniprotKb protein accession number and primary name, keyword annotation, NCBI taxonomy (taxon name/species name) or publication PMID are accepted. The second way to retrieve PPIs is the sequence search: here, the primary sequence of a protein is launched against the VirHostNet interacting protein sequences database. 

Virus/host PPIs can also be browsed according to the taxonomy lineage of viruses or to UniProtKB keyword annotation. Finally, prediction and the visualisation of virus-virus and virus-host protein-protein interactions can be obtained via the Interology web service. VirHostNet 3.0 provides a tutorial on how to use Interology [94]. 

In the most recent work citing this tool, the authors investigated the alternative splicing alterations induced by influenza A virus infection of the human alveolar A549 cells. To achieve the goal, RNAseq data were analyzed and made accessible for browsing through a user-friendly interface [96].

##### HPIDB

Current release 3.0 of the Host–Pathogen Interactions (HPI) database (HPIDB) [97] contains almost 70,000 manually curated entries. HPIDB is a member of the IMEx consortium, meaning that the annotations provided by its curators meet community standards for providing detailed contextual experimental information and facilitating data sharing. Moreover, this database integrates HPI data from existing external sources and contains tools to infer additional HPIs where annotated data are scarce [98]. 

Gene ontology functional information and implementation of network visualisation enhance the performance of this tool. HPIDB 3.0 provides sets of HPIs by inhouse manual curation of published, experimental HPI data and bringing in external HPI data provided by twelve existing molecular interaction resources. 

Manually curated HPI can be used directly for network analysis and to improve the accuracy of HPI computational prediction. Indeed, experimentally derived HPI data come with information for network modelling and detailed experimental information that supports a full assessment of the biological context of the interactions [98]. 

HPIDB 3.0 makes use of IntAct annotation. HPI files contain the minimum information required to visualise HPI networks and display accession of the pathogen protein, accession of the interacting host protein, interaction detection method and the publication reporting this interaction. Moreover, host and pathogen taxonomy identifiers, protein names and the molecular interaction type are reported to facilitate searching of these files as well as standard information regarding the annotator and timeline for the annotation. All interaction data available in HPIDB 3.0 are freely available for download [98].

The authors of a very recent study reported a comprehensive map of the Epstein–Barr virus (EBV)–human protein interactions, set up by combining data from HPIDB 3.0 with curated high-throughput proteomic data from the literature. In addition to shed more light on EBV-host interactions, this study [99] opens the route to drug discovery projects and therapeutic interventions.

##### Viruses.STRING 

Viruses.STRING is a protein–protein interaction database tailored to virus–virus and virus–host interactions [100]. The use of the CytoscapeSTRING app is useful to improve the analyses with the STRING Viruses website [101]. This latter allows three kinds of protein searches: (i) a complete set of proteins in a virus, (ii) a single protein in a virus, and (iii) multiple proteins in a virus. 

Interactions between virus and host proteins are visualised in a network along with the most strongly interacting host proteins. Here, the nodes in the network are coloured in brick red (viral proteins) and slate blue-green (host proteins). By clicking on the nodes or edges, information about the proteins and their contribution to the interaction is available. Both virus and host orthology relations were taken from EggNOG 5.0 [102]. Since the generic STRING database transfers a known interaction between two proteins to orthologous proteins from other species, the same happens with virus–virus PPIs [103].

In recent work, gene interaction networks between the human respiratory syncytial virus (HRSV), human metapneumovirus (HMPV) respiratory pathogens and their host were evaluated [104]. These two viruses are the leading causes of upper and lower respiratory tract infections in non-immunocompetent subjects. The Viruses.STRING database was used for the identification of the host–viruses interaction networks due to a lack of knowledge with respect to the gene interaction networks between the two viruses and their host. The results of Viruses.STRING analysis was depicted as a Venn diagram.

##### HCVpro

HCVpro is a free database of PPIs for manually verified hepatitis C virus-virus and virus-human protein interactions from literature and databases [105]. This database is enriched with information on genes related to hepatocellular carcinoma and corresponding proteins. Incorporated proteins have been mapped onto Gene Ontologies, canonical pathways, OMIM and extensively cross-referenced to other essential annotations. The structure and functions of HCV proteins are reported, as well as the current state of drug and vaccine development and links to articles. The database can be queried using protein IDs, chromosomal location of a gene, interaction detection methods, indexed PubMed sources and HCVpro/BIND/VirusMint IDs. 

Although the original paper [105] HCVpro was stated to be available at two websites, unfortunately, at the time of writing, they are offline. 

##### PHISTO

The PHISTO database aims to facilitate HPI studies that provide potential therapeutic targets for infectious diseases [106]. PHISTO integrates tools for the visualisation of HPI networks, graph–theoretical analysis of targeted human proteins, BLAST search and text mining for detecting missing experimental methods. HPIs are imported from different databases. To present protein interaction data in a consistent format, UniProt IDs and names of interacting proteins, taxonomy IDs and names of pathogens, experimental methods and PubMed IDs of literature references are collected.

The web interface [107] is user-friendly and allows the carrying out of a “Quick Search” (without a specified identifier) or an “Advanced Search” based on any selected subset of identifiers (e.g., taxonomy ID and name of pathogen, UniProt ID and name of pathogen protein, UniProt ID and name of human protein, experimental method and literature reference).

Moreover, the “Browse” function lets the user access all HPI data of any specified pathogen within the taxonomical classification. Output data are displayed as an HPI network in a bipartite graph fashion, allowing users to capture HPI mechanisms. Statistics of search results can be visualised through pie or bar charts. Finally, the “Graph Analysis” tool embedded in PHISTO can give crucial insights into the attacking strategies of pathogens during the infection [106].

PHISTO was used to provide a general overview of infection strategies used by different pathogens [108]. This work shows that Adenoviruses, HIV, Papillomaviruses, and Polyomaviruses are observed to target one or more proteins in each of four groups: cell cycle proteins, transcription factors, apoptosis regulators, and nuclear membrane proteins. Proteins of Hepatitis viruses interact with PTMA, EP300, TAF1, and p53, while proteins of Herpesviruses interact with PTMA and SUMO1. On the other hand, viral groups of Influenza, Puumala, Tula, SARS, and Vaccinia are observed to target nuclear membrane proteins [108].

##### HVIDB

HVIDB (human–virus PPI database) [109] offers two levels of analyses by combining multiple human–virus PPI data resources and predicting interactions between human and viral proteins. This database is enriched with experimentally derived PPIs and three-dimensional (3D) structures of PPIs (from PDB or predicted by using homology modelling of protein complexes), virally targeted human complex information retrieved from CORUM [110,111] and hu.MAP [112,113] and manually collected host factor data. Moreover, this resource harbours interactions with differential expression information of human genes post-viral infections, human tissue-specific gene expression profiles and functional enrichment analysis of virus-targeted human proteins. 

**Figure 1:**
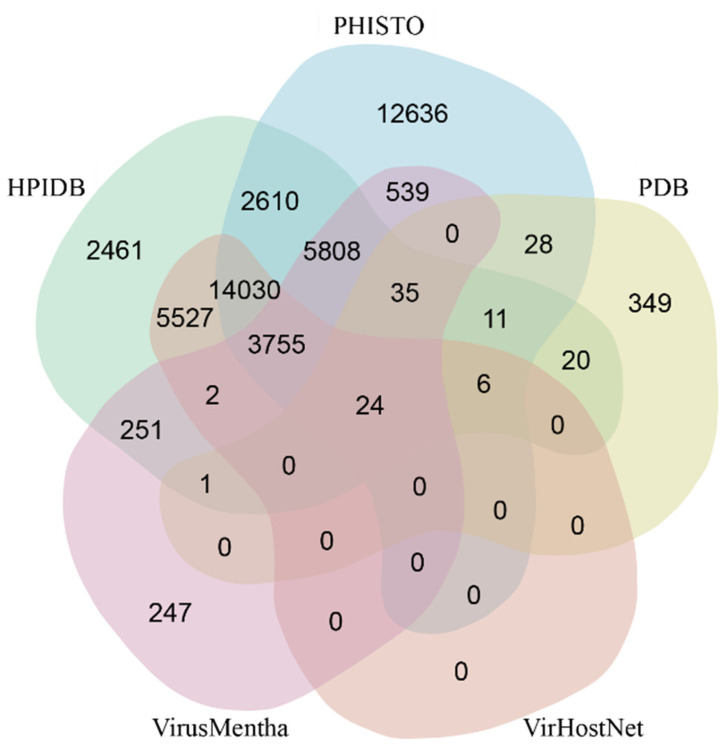
Experimentally validated PPIs in HVIDB taken from the shown public databases, and dataset overlapping. This image was taken as a snapshot from the HVIDB website [109], with permission from Yang et al. [114].

Experimentally verified PPIs are taken from public databases (i.e., HPIDB, PHISTO, VirHostNet, Virus-Mentha and PDB, see Figure 1) and recently published literature. 

Users are allowed to access the HVIDB data via different searching/browsing modules. Entries can be searched through UniProt ID, gene name and protein name or PPI data, and associated auxiliary information can be retrieved by browsing through corresponding lists, covering human–virus PPIs, 3D complex structural information, virally targeted human complexes, differential expression information and host factors. 

HVIDB provides two individual modules for accessing differentially expressed genes (DEGs) post-viral infections and virally targeted human complexes. Moreover, users can provide a pair of human and viral proteins in FASTA format to predict the presence of an interaction between them [114].

## 3. Phylogenetics and Phylodynamics Tools

Comparison of homologous proteins plays a key role in understanding the phylogeny of the proteins themselves and in reconstructing the ancestral proteins from which the modern molecules evolved [115,116]. The large amount of data made available from aforementioned databases, in addition to modelling studies, allows researchers to perform phylogenetic studies on both genomic and amino acid sequences. This can help understand the origin and dynamics of viral outbreaks, drivers of spatial spread, characteristics of transmission clusters and factors that contribute to enhanced viral pathogenicity and adaptation [117].

All the genetic changes accumulated by viruses, if put in relation to the time scale of the respective spread events, provide us with a general picture of the viral evolutionary process and lead to the definition of viral phylodynamics [118]. Furthermore, due to the high speed of this process, several mutations can occur simultaneously with geographical dispersal, and the overriding interaction characterises a spatial phylodynamic process, which can be recovered from genomic data using phylogeographic analyses [119]. These new disciplines also require more complex visual representations to support the projection of viral spread onto a geographical map, leading to the development of visualisation tools to better represent the phylogenetic trees, enriched with many other important aspects, such as population size dynamics over time, transmission networks and estimates of ancestral states for traits of interest [120].

### 3.1. RAxML

RAxML (Randomised Axelerated Maximum Likelihood) stands as a cornerstone in the field of phylogenetics, offering a robust framework for reconstructing evolutionary relationships based on molecular sequence data. Developed as AxML at first [121], this software utilises a Maximum Likelihood (ML) approach [122] to infer phylogenetic trees, leveraging the speed and efficiency of randomised heuristics to explore vast tree spaces. Since its inception, RAxML has undergone significant evolution, marked by successive versions that have introduced enhanced algorithms, improved computational efficiency, and increased accuracy in phylogenetic inference. The last available version is RAxML-NG [123], an upgrade of RAxML v8 [124], which can be downloaded from GitHub [125]. 

With each version, RAxML has solidified its reputation as a versatile and reliable tool in the phylogenetic toolkit, catering to the evolving needs of the scientific community engaged in molecular evolutionary studies. In a 2023 paper from Legason and colleagues, RAxML has been used to analyze the linkage between Epstein–Barr virus (EBV) and endemic Burkitt Lymphoma (eBL), providing the first-ever comprehensive mutational profile of EBV in eBL clinical samples, looking at the phylogeography of different strains [126]. In the same year, RAxML v8 was used to build a phylogenetic tree of the hepatitis delta virus currently circulating in Lazio, Italy [127].

### 3.2. BEAST

BEAST (Bayesian Evolutionary Analysis Sampling Trees), now in its 1.10.4 version [128], is a versatile cross-platform programme including a core set of software designed for Bayesian analysis of molecular sequences utilizing the Markov Chain Monte Carlo (MCMC) method [129]. It represents the state of the art in phylodynamic tools scenario, with a primary focus on the inference of rooted, time-measured phylogenies through the application of strict or relaxed molecular clock models, in addition to being a tool for phylogenetic reconstruction.

BEAST also acts as a robust framework for empirically testing evolutionary hypotheses, thus avoiding the need for reliance on a single tree topology. Adopting MCMC, BEAST can conduct a complete exploration of the tree space, assigning weights to each tree in accordance with its posterior probability.

In a very recent work, Jonathon D. Gass and colleagues used BEAST for molecular clock analysis on sequences of H5Nx high pathogenicity avian Influenza (HPAI) viruses—which caused the 2016 outbreak in North America and Europe—obtained from samples collected during the 2016–2022 period [130].

Looking at a large-scale use of this software, both in terms of timescale and number of sequences, Lu et al. (2024) [131] demonstrate the application of BEAST to estimate the phylogenies of human infectivity and transmissibility across 1408 genome sequences from 743 distinct RNA virus species. This comprehensive analysis explores temporal changes in viral diversity, highlighting significant evolutionary trends in RNA viruses over extended periods.

**Figure 2:**
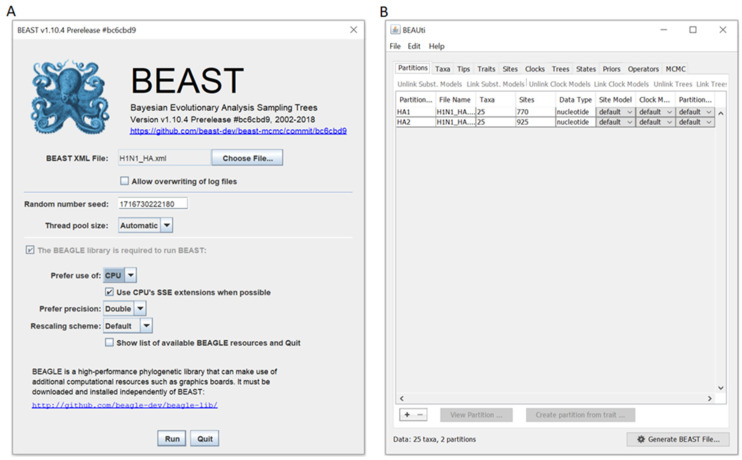
Graphical interfaces of (**A**) BEAST v1.10.4 and (**B**) BEAUti, both of which are included in the available package. The first step is to import the sequence alignment (FASTA or NEXUS format) using BEAUti. The programme then allows users to generate a sorted BEAST XML file. The XML file parameters can be set directly from the BEAUti interface, enabling the user to configure various settings, including substitution matrix model, clock model, tree model and prior, ancestral state reconstruction, and MCMC parameters. Once the XML file is created, it can be uploaded to BEAST, which can be run in default mode or with modified parameters. Images are taken from the examples package of the program, which can help practising with the software. Data from Suchard et al. [128].

The full package is available at the BEAST community web site [132], and the latest version requires the presence of the BEAGLE library [133], available on the web [134], with guided steps to the installation. The BEAST application features a graphical interface that allows the users to run the programme using default or modified settings (Figure 2A). 

Noteworthy, the input file format must be XML, which is why the package includes the BEAUti application (Figure 2B), which is essential to import sequences files (FASTA, NEXUS and BEAST) and generate structured XML files that can be run with BEAST.

In addition to the core software package, different programs are distributed separately but are an important part of the BEAST tool kit. FigTree (v1.4.4) [135] works as a graphical viewer for phylogenetic trees, doubling as a tool for crafting publication-ready figures. Initially created to fulfil Rambaut’s personal needs, it may lack the refinement of commercial software, but its primary focus on displaying summarised and annotated trees produced by BEAST is well achieved. Another visualisation software package related to BEAST is Tracer [136], which is designed to process MCMC trace files containing parameter samples and to interactively explore the high-dimensional posterior distribution.

The BEAST family also includes the SPREAD software [137] and its latest version, SpreaD3 [138], implementing JavaScript libraries (e.g., D3). SPREAD maps phylogenies with spatial-temporal data and exports summaries to Keyhole Markup Language (KML) for animated and interactive diffusion visuals, incorporating Bayes factors calculation for historical diffusion hypotheses. 

It is worth noting that on the web, BEAST 2 [139] is also available, which originates as an independent project and employs a modular approach, allowing users to customise the software according to their specific needs. However, it is important to acknowledge that this process may be somewhat challenging for individuals lacking certain programming skills. 

### 3.3. IQ-TREE

IQ-TREE (available at [140]) stands at the forefront of phylogenetic software, renowned for its statistical rigour and iterative refinement. Developed to address the limitations of traditional ML approaches [122], IQ-TREE incorporates sophisticated models of sequence evolution and performs an iterative optimisation process to enhance the accuracy of phylogenetic tree inference [141]. 

The software distinguishes itself by employing a broad range of statistical tests like the Ultrafast bootstrap approximation (UFBoot) to assess branch support analyses and model fit [142]. With each subsequent release, the software has seen notable improvements, refining its optimisation algorithms, expanding model selection capabilities, and incorporating advanced statistical techniques. 

**Figure 3:**
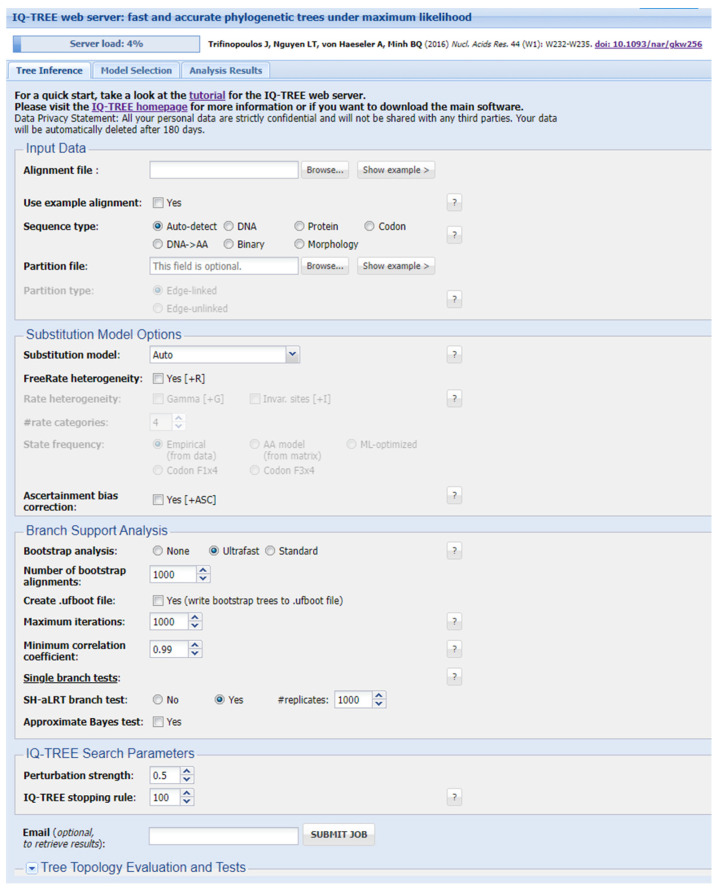
Graphical interface of the IQ-TREE web server [145]. Aligned sequence files (PHLIP, FASTA, NEXUS, CLUSTAL, or MSF format) can be uploaded using the Browse command. The default settings allow the programme to automatically detect the input sequence type and determine the best-fit substitution model, though users can also manually select from different models. Manual selection unlocks the ability to choose all provided common rate heterogeneity models across sites. The authors suggest using the FreeRate model [148]. If the input sequences do not contain constant sites, the ascertainment bias correction model can be included [149]. Branch support analyses enable users to include bootstrap analyses; the default setting uses the ultrafast bootstrap approximation (UFBoot) [142]. All default values are set to the maximum allowed and can be left as preset. The default single branch test is the SH-aLRT branch test [150], but users can also include the approximate Bayes test [151]. The default search parameters generally perform well but may not be suitable for all datasets. If tree search issues persist, the authors recommend repeating the analysis with at least 10 runs, adjusting the perturbation strength and stopping rule settings, especially for datasets with many short sequences. It is possible to find more information on the settings, including references, by pressing the “?” button next to the respective parameter. Data from Trifinopoulus et al. [144].

The latest version, IQ-TREE 2 [143], showcases heightened computational efficiency and an expanded suite of models, underscoring IQ-TREE’s commitment to providing researchers with a high-performance tool for rigorous and statistically sound phylogenetic analyses. Moreover, the web tool W-IQ-TREE [144] is available online [145], simplifying the usage of the IQ-TREE software via a graphic interface (Figure 3), providing a maximum of 1 GB RAM for the computational work. 

The relevance of this tool to virology is supported by several studies; for example, in 2021, Sulaiman et al. [146] combined IQ-TREE and BEAST to analyze the zoonotic potential of H9N2 AIV in live bird markets in Nigeria, emphasising warning about this practice within the presence of co-circulating AIV. In very recent work, IQ-TREE has been used to perform phylogenetic analysis on the full-length spike gene of porcine epidemic diarrhoea virus variant ECQ1 circulating in China (2017–2019), highlighting its role as a potential live vaccine candidate [147]. 

### 3.4. Nextstrain

Nextstrain [56] is an innovative programme for real-time monitoring of pathogen evolution. Based on genomic data, the framework incorporates phylogenetic and visualisation approaches to track the spread and evolution of pathogens such as viruses, also providing a wide range of geographical information for accurate phylodynamic analyses. Nextstrain offers an accessible and interactive platform, enabling scientists to monitor the genetic evolution of pathogens in real-time, with significant implications for epidemiological surveillance and the design of disease control strategies. 

The website [55] makes available multiple datasets of different viruses (influenza, SARS-CoV-2, RSV, etc.), which are constantly updated by Nextstrain community. It also incorporates the last version of IQ-TREE [143]. The Nextstrain package has been used to demonstrate changes in the origins of resurgent HPAI H5, revealing significant shifts in virus ecology and evolution [152]. In the same year, Focosi and Maggi used Nextstrain to emphasise the issue of unregulated taxonomy characterisation of SARS-CoV-2 variants worldwide, depending on classification criteria adopted among various countries and organisations to update databases [153].

### 3.5. PhyloGeoTool

PhyloGeoTool [154] is an application (free download from [155]) facilitating interactive exploration of large phylogenies and associated clinical data. This tool is based on an algorithm for automatic phylogeny partitioning into optimal clusters, refining each through recursive processes. The software needs to be installed locally; however, it is accessible through a web browser. 

Due to its web app nature, operating this programme requires some expertise. Beyond this, the interface provides users with a concise view of the cluster tree, presenting detailed information on sequence attributes, geographic dispersal, and user-selected attribute distributions. Hovering over a cluster activates a specific bar chart for attribute insights. Noteworthy, PhyloGeoTool only requires a phylogenetic tree and attribute data for each taxon, eliminating the need for a database structure. 

To highlight PhyloGeoTool’s potential, Lubin and colleagues presented a case study concerning the transmission of HIV-1 drug resistance in Europe. They investigated the prevalence of transmitted drug resistance and its association with geography, HIV-1 subtype and clades in the phylogenetic tree [154].

### 3.6. MEGA

The Molecular Evolutionary Genetics Analysis (MEGA) software (available from [156]) employs a comprehensive approach to phylogenetic reconstruction, integrating various methods such as ML and Neighbor-Joining [157]. Since its initial release [158], MEGA has undergone substantial improvements, enhancing its functionality and usability. Indeed, the latest version, MEGA 11 [159], in addition to retaining the robust suite of analytical tools from its predecessors, introduces novel functionalities, incorporating powerful statistical methods for the analysis of complex datasets and the ability to handle large-scale genomic data. 

**Figure 4:**
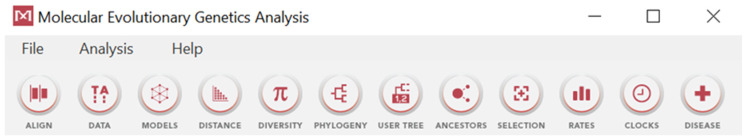
Launch bar of the graphical (GUI) version of MEGA 11 [156].

Noteworthy enhancements include improved algorithms for tree inference, more accurate estimation of evolutionary distances, sophisticate models of sequence evolution, and a user-friendly interface that makes the usage of the different proposed tools extremely intuitive (Figure 4). 

The continuous development and refinement of MEGA make it an indispensable tool for researchers and scientists engaged in evolutionary virology and molecular phylogenetics. This tool was used to reconstruct the phylogeny of SARS-CoV-2 after two years of spread, building a phylogenetic tree of the virus from the first Wuhan strain [160]. 

MEGA also proved helpful in an investigation on the type B DNA polymerase gene of the African Swine Fever Virus (ASFV)—aimed at understanding the genetic diversity of that pathogen—by purposing a five-clades classification of the entire *Asfarviridae* family and highlighting the importance of phylogenetic tools [161].

The available options allow the user to perform various actions:

- ALIGN: allows the alignment of protein and genomic sequences using one of the algorithms, ClustalW [162] or MUSCLE [163];
- DATA: users can import and explore datasets of sequences in different formats through this option;
- MODELS: enables the construction of various mutation models, with possible automatic selection of the best algorithm;
- DISTANCE: allows the calculation of pairwise or overall mean distances within sequence datasets;
- DIVERSITY: enables the calculation of the diversity within an entire group or a previously defined subgroup;
- PHYLOGENY: users can build phylogenetic trees from the downloaded sequences using methods such as NJ or ML, possibly adding bootstrap analysis;
- USER TREE: allows the submission and analysis of existing tree files using different methods like ML or Parsimony;
- ANCESTORS: infers ancestral sequences from a tree file using ML or parsimony;
- SELECTION: performs analyses on SNPs, including Tajima’s test of neutrality [164];
- RATES: calculates the nucleotide substitution rates in sequence alignments, using position-by-position rates (ML) or Gamma rates [165];
- CLOCKS: allows to compute a timetree using the RelTime method [166] or to perform tests like the molecular clock test (ML) or the Tajima’s relative rate test [167];
- DISEASE: assesses the impact of non-synonymous mutations on health using MEGA Molecular Diagnoses, with the possibility to access pre-computed diagnoses.

Notably, by clicking on the “Help” command, users can open the guided tutorial within the manual document. Courtesy of Kumar et al. [168].

### 3.7. Virus Pop

Last but not least, Virus Pop [169] is a recent pipeline for adding new branches to a protein phylogenetic tree (available at [170]). The idea behind this programme is that metagenomics tools in virology, although crucial for identifying unknown viruses, are unfortunately limited in their evaluation due to the reliance on datasets with known viral sequences. 

To improve the programme’s capacity to identify distant viruses, simulating realistic evolutionary directions is essential. Expanding databases with realistic simulated sequences could enhance alignment-based strategies for detecting distant viruses. Leveraging tools from the latest version of IQ-TREE [143], Virus Pop simulates protein evolution realistically, considering site-dependent substitution rates in the input dataset.

Virus Pop offers options to simplify the construction of the starting dataset, and configurable steps allow users to precisely constrain the locations of the generated sequences within the tree. As a result, a set of simulated sequences complements the initial tree with new branches connected at specified topological positions. Users can either automatically set evolutionary distances based on observed distances in the starting dataset or manually specify them. 

To support the validation of Virus Pop, the authors tested its software on Sarbecovirus spike protein and Circovirus capsid protein, demonstrating the accuracy of prediction [169]. 

## 4. Conclusions

The study of viruses has been enormously enhanced by revolutions in molecular biology and genomics, i.e., since it became possible to rapidly obtain the sequences of entire viral genomes and transcriptomes. At the same time, this has generated huge amounts of data and, therefore, the need to deposit, analyze and make such information available to the scientific community. 

Comparing the pioneering times of omics sciences and bioinformatics with current research trends, we have moved from a phase in which efforts were concentrated on the production and archiving of “missing sequences” to the actual need to filter the “noise” associated with redundancy and/or incorrect data that may hide high-quality, real information or result in misleading suggestions. 

Indeed, “big” does not always correspond to “good”, and this is even more true if we consider that, from big, data have become “huge”, as illustrated previously. Therefore, it is time for the scientific community to move away from its a priori veneration of “big data” and begin coordinating efforts to reduce noise in the data. This can be achieved, for example, by eliminating the excess of useless predictions overlapping with real data, encouraging the integration of database entries through processes supervised by experts and, above all, by transforming many databases—very useful but still “raw”—into “knowledgebases” with more secure, complete and quality information.

This paper focused on databases and, in particular, those of interest to the virology community, with a special focus on interactions databases, as well as phylogenetics and phylodynamics tools. 

Table 1 summarises databases/tools for virus research presented here, with key features, corresponding citations and website links. 

However, the great availability of sequences and the great progress in the field of functional characterisation and inference have led to the development of a large number of tools for functional analyses, which will be covered in the next and second article of this series: *Bioinformatics goes viral: II. sequence-based and structure-based functional analyses for boosting virus research*.

## Figures and Tables

**Table 1:** Databases, tools and web portals for virus research presented in this article. PPI, protein-protein interaction. A summary of their key features is reported, followed on the right side column by numbers (according to citations along the text) that correspond in the reference list to website URLs for online working (or for free download).

Name	Type	Key Features	Website
Pathogens	web portal	nucleotide sequences, raw genomic data, sample metadata, scientific literature on HIV, influenza, Hepatitis B, *P. falciparum* and lesser-known pathogens affecting humans	[27]
ViMIC	database	information on virus mutation sites, viral integration sites, viral target genes, >1,000,000 sequences of eight viruses in almost 80 human diseases, cis-effects of virus–host interactions	[42]
VirusDB	database and tool	virus classification by closest neighbour genome search	[44]
IVDB	database	analysis platform for genetic, genomic, and phylogenetic studies of the influenza viruses	[46]
IVDT	tool	shows the worldwide geographic distribution of chosen viral genotypes and couples genomic data with epidemiological data	[47]
Los Alamos HIV databases	web portal	information available in eight sections: Sequence DB, Immunology DB, HXB2 Feature DB, Env Feature DB, Neutralisation DB, HCV DB, HFV DB, and COVID-19 Genome Analysis Pipeline. Online tools for alignment, genome navigation, annotation, consensus building, motif scanning, peptide mapping, heat maps	[49]
ViralZone	web portal	allows you to navigate in five main sections (Taxonomy, Biology, Hosts, Genomes, Re-sources)	[51]
GISAID	database	Sections: EpiFlu™ (influenza virus), EpiCoV™ (SARS-CoV-2), EpiPox™ (MPXV), EpiArbo™ (Arbo-viruses)	[52]
BV-BRC	web portal	unified interface for sequence and data mining, and a powerful suite including several command-line bioinformatic tools for bacterial and viral research	[63]
BioGRID	database	hosting protein, genetic and chemical interactions from different species	[70]
IntAct	database	general PPI database, including information on viruses	[78]
MINT	database and tool	general PPI database, including information on viruses, with VirusMINT search tool	[84]
VirusMentha	database	detects all published virus–host PPI integrating other databases	[87]
VirHostNet	database and tool	modelling of host/virus interactome using an undirected graph for data exploration and examination	[95]
HPIDB	database	contains almost 70,000 manually curated Host–Pathogen Interaction (HPI) entries with gene ontology functional information	[97]
Viruses.STRING	database and tool	database tailored on virus–virus and virus–host interactions, with the CytoscapeSTRING app	[101]
HCVpro	database	manually verified hepatitis C virus–virus and virus–human protein interactions; proteins are mapped onto Gene ontologies, canonical pathways, OMIM, and extensively cross-referenced	[105]
PHISTO	database and tool	integrates tools for the visualisation of HPI networks, graph–theoretical analysis of targeted human proteins, BLAST search and text mining	[107]
HVIDB	database	combines multiple data resources for predicting human–virus PPIs; enriched with experimentally derived PPIs and 3D structures of PPIs (from PDB or predicted by using homology modelling of protein complexes)	[109]
RAxML	tool	evolutionary relationships reconstruction based on a Maximum Likelihood (ML) approach	[125]
BEAST	tool	versatile cross-platform software suite for Bayesian analysis via Markov Chain Monte Carlo method	[132]
IQ-TREE	tool	incorporates sophisticated models of sequence evolution and performs an iterative optimisation process to enhance the accuracy of phylogenetic tree inference	[140]
Nextstrain	tool	programme for real-time monitoring of pathogen evolution	[55]
PhyloGeoTool	tool	facilitaties interactive exploration of large phylogenies and associated clinical data based on automatic phylogeny partitioning into clusters refined through recursive processes	[155]
MEGA	tool	Molecular Evolutionary Genetics Analysis software integrating various methods such as ML and Neighbour-Joining. It also incorporates powerful statistical methods	[156]
Virus Pop	tool	pipeline for adding new branches to a protein phylogenetic tree	[170]

## Data Availability

Not applicable.
